# The Interplay Between Adipokines and Body Composition in Obesity and Metabolic Diseases

**DOI:** 10.7759/cureus.78050

**Published:** 2025-01-27

**Authors:** Umesh Zadgaonkar

**Affiliations:** 1 Department of Nutrition and Dietetics, Global University, Itanagar, IND

**Keywords:** adipokines, insulin resistance, leptin, metabolic diseases, obesity

## Abstract

The worldwide health system faces challenges from obesity and related metabolic disorders because they exhibit both rising rates of occurrence and intricate pathophysiological mechanisms. This work examines how adipokines interact with body composition during obesity to control important metabolic functions. Bioactive molecules produced by adipose tissue function as adipokines which regulate essential biological pathways that control inflammation response and insulin sensitivity alongside energy balance management and immune system operation. The disruption of adipokine secretion and function leads directly to metabolic disorders which include insulin resistance and persistent inflammation characteristic of obesity-related conditions. This article investigates the therapeutic possibilities of adipokine pathway manipulation through new pharmacological approaches and lifestyle changes alongside personalized medicine developments. Researchers analyze adipokines as important biomarkers for patient disease classification and their application in creating individualized treatment plans. The review highlights existing research deficiencies and obstacles that stand in the way of applying adipokine discoveries to clinical settings. This article integrates existing research to show how adipokine regulation helps prevent obesity-related metabolic issues and suggests directions for future studies to enhance treatment results.

## Introduction and background

Obesity is a multifactorial and complex condition in which adipose tissue is excessively accumulated and is associated with many chronic diseases. It is not only a cosmetic problem, but rather it is a serious public health problem of major significance for morbidity and mortality. Worldwide, obesity is on the rise as does its prevalence as a leading risk factor for metabolic diseases (Type 2 diabetes mellitus (T2DM), cardiovascular disease, and some types of cancer). According to the World Health Organization, 2022, “One in eight people had obesity, adult obesity has more than doubled since 1990, while adolescent obesity quadrupled, globally. In that year, 2.5 billion adults were overweight and 890 million were obese. In addition, 43% of adults were overweight, and 16% were obese. Thirty-seven million children under the age of 5, 390 million children, and adolescents aged 5-19 are classified as overweight, including 160 million who are categorized as having obesity, and childhood obesity is also increasing” [1]. The increase in this alarming trend has serious underlying consequences and provoked the urgency to tackle them. These mechanisms represent complex relations among genetic, environmental, and lifestyle factors that cause the disturbance of normal metabolic function, causing insulin resistance, altered lipid metabolism, and chronic inflammation [2].

In this case, obesity also facilitates the development of a spectrum of comorbidities, including "hypertension", "dyslipidemia", and "NAFLD" (non-alcoholic fatty liver disease), which, together with that risk, increase cardiovascular risk. This increasing prevalence of these diseases emphasizes the importance of having a greater understanding of how excess adipose tissue and metabolic dysfunction are somehow linked [3].

Adipose tissue functions as an active endocrine organ because it produces bioactive molecules known as adipokines beyond its primary role as an energy storage system. These adipokines function as essential regulators for fundamental metabolic processes which encompass glucose balance as well as lipid metabolism together with energy regulation and immune responses [4]. The most widely known adipokines are leptin, adiponectin, and resistin each of which has a unique but overlapping role in maintaining metabolic balance. For example, leptin, the main mediator of the control of appetite and energy balance, signals the brain about the body's energy stores, whereas adiponectin protects against insulin resistance and has remarkable anti-inflammatory properties [5]. On the other side, resistin has been linked to increased inflammation and insulin resistance [6].

These adipokines show dysregulated secretion and activity patterns which lead to metabolic disorders related to obesity including insulin resistance which represents the fundamental characteristic of T2DM. The worldwide increase in obesity rates demonstrates the growing importance of adipokines because they drive metabolic disorder pathophysiology and boost chronic disease risks including diabetes and cardiovascular conditions along with inflammation [6]. In obesity, adipose tissue changes structurally and functionally; this leads to imbalances in adipokine release, leading to insulin resistance and persistent low-grade inflammation. Elevation in proinflammatory adipokines for example TNF α and IL 6 is seen in obese people, and this contributes to systemic inflammation leading to further worsening of metabolic disturbances. On the other hand, individuals with obesity or metabolic syndrome often exhibit low levels of adiponectin, a molecule that normally protects against inflammation and insulin resistance [7]. Since adipokines play critical roles in metabolic regulation and are linked to obesity-related diseases, adipokines have become targets for therapeutic intervention [4]. New treatment strategies for obesity and its comorbidities require understanding of adipokine secretion and its influence on various metabolic pathways. The functions of these important adipokines in obesity and metabolic diseases have been addressed in this review.

The objective of this review is to understand the intricate interaction between adipokines and body composition during obesity and metabolic disease. Changes in body fat distribution modify adipokine profiles and how adipokines in turn influence body composition and metabolic health are evaluated. We synthesize recent findings to understand how adipokines are bidirectionally related to body composition and potential therapeutic targets to alleviate metabolic dysfunctions associated with obesity.

## Review

Adipose tissue: an endocrine organ

Adipose tissue, which has long been thought of as an inactive organ for storing energy, has recently been identified as a dynamic endocrine organ involved in metabolic regulation.

In general, adipose tissue was once considered merely a triglyceride depot, a place where triglycerides were stored to be mobilized when calories were insufficient. The discovery of leptin in 1994, an adipocyte-secreted hormone that controls appetite and energy balance, shifted this perspective further with the discovery of adipose tissue's endocrine functions [8]. Since then, numerous adipocyte-derived hormones (collectively referred to as adipokines) that affect several physiological processes involving insulin sensitivity, inflammation, and lipid metabolism have been identified [7].

Secretion of adipokines and their systemic effects

These adipokines show dysregulated secretion and activity patterns which lead to metabolic disorders related to obesity including insulin resistance which represents the fundamental characteristic of T2DM. The worldwide increase in obesity rates demonstrates the growing importance of adipokines because they drive metabolic disorder pathophysiology and boost chronic disease risks including diabetes and cardiovascular conditions along with inflammation.

Adipocytes secrete a variety of adipokines that exert systemic effects (Table 1).

**Table 1:** The secretion of adipokines and their systemic effects

Adipokine	Primary Function	Systemic Effects	Reference
Leptin	Signals satiety to the hypothalamus, suppressing appetite, and regulates energy intake and expenditure	Suppresses appetite, regulates energy balance, and influences metabolism.	[8]
Adiponectin	Improves insulin sensitivity and has anti-inflammatory qualities	Protects against metabolic disorders by enhancing insulin sensitivity and reducing inflammation.	[9]
Resistin	Plays a role in insulin resistance and inflammatory events	Contributes to insulin resistance and inflammation, though its exact role in humans is not fully understood.	[10]

Adipose tissue: white and brown

There are various types of adipose tissue, primarily brown and white adipose tissue (WAT and BAT), each with special characteristics and uses.

WAT (White Adipose Tissue)

WAT is made up of unilocular adipocytes including a single large lipid droplet and is the principal site for energy storage. In addition, it is an endocrine organ that produces different adipokines that regulate metabolism [11,12].

Brown Adipose Tissue (BAT)

Adipocytes that are multilocular have a high concentration of uncoupling protein-1 (UCP1) containing mitochondria and many tiny lipid droplets. BAT specializes in oxidizing fatty acids and contributing to energy expenditure through thermogenesis [13].

Adipokines in obesity and metabolic diseases

Adipokines, which are adipose tissue secreting signaling molecules, are important for maintaining metabolic homeostasis. The adipokines are dysregulated in obesity and its associated metabolic disorders (Table 2).

**Table 2:** Key adipokines and their impact on metabolic diseases

Adipokine	Primary Function	Dysregulation in Obesity	Clinical Implications	Reference
Leptin	Appetite regulation and energy expenditure	Elevated levels with reduced hypothalamic response (leptin resistance)	Leptin resistance contributes to obesity; targeting leptin signaling may help regulate appetite and improve weight management.	[14]
Adiponectin	Improves insulin sensitivity, anti-inflammatory	Reduced levels, contributing to insulin resistance	Low adiponectin levels are linked to metabolic disorders; increasing adiponectin could enhance insulin sensitivity and reduce inflammation.	[15]
Resistin	Related to inflammation and insulin resistance	Increased levels, related to chronic inflammation	Elevated resistin levels are associated with insulin resistance; targeting resistin could help manage inflammation and improve insulin function.	[16]
Visfatin	Modulates glucose metabolism	Elevated, with an unclear role in insulin resistance	Elevated visfatin may influence glucose metabolism; further research is needed to clarify its potential as a therapeutic target for insulin resistance.	[17]
Omentin	Enhances insulin action, anti-inflammatory	Decreased levels in obesity and metabolic syndrome	Omentin levels are lower in obesity and metabolic syndrome; increasing omentin could improve insulin sensitivity and reduce inflammation.	[18]
Chemerin	Regulates adipogenesis and inflammation	Increased levels in metabolic syndrome, T2DM, and obesity	Elevated chemerin levels are associated with obesity and metabolic diseases; targeting chemerin may reduce inflammation and improve metabolic health.	[19]

Leptin: regulator of appetite and energy balance

A key regulator of energy balance is leptin, a predominantly white adipose tissue-secreting hormone. It tells the hypothalamus to keep us from overeating and burn more calories. However, in obesity, elevated levels of leptin cannot trigger these effects because leptin has become "leptin resistant", adding to further weight gain and metabolic dysregulation. These studies demonstrated that a major factor is leptin's inability to cross the blood-brain barrier in obese individuals [14].

Adiponectin demonstrating anti-inflammatory and insulin-sensitive properties

Adiponectin is the only adipokine whose levels decrease with increasing obesity. It increases the sensitivity of skeletal muscle and liver insulin to transport glucose across the cell surface and eliminate fatty acids. Adiponectin also has anti-inflammatory properties, opposing the actions of proinflammatory cytokines, such as TNFα, which are increased in obesity. In obesity, adiponectin is reduced; this increases the risk of T2D and cardiovascular disease and exacerbates insulin resistance [15].

Resistin linked to insulin resistance and inflammation

Insulin resistance and chronic inflammation have been linked to resistin released by adipocytes. In humans, macrophages within adipose tissue secrete resistin rather than adipocytes. In obese individuals, elevated resistin levels correlate with increased inflammatory markers and decreased insulin sensitivity [16]. However, the exact ways that resistin influences glucose metabolism are unknown.

Other emerging adipokines: roles and clinical implications

Recent years have seen the identification of novel adipokines with significant roles in metabolic regulation.

Visfatin

Visfatin was initially thought to act like insulin, and it regulates glucose metabolism and is elevated in obesity. Nevertheless, there is conflicting data about its exact function in metabolic diseases [17].

Omentin

Omentin is secreted primarily by visceral adipose tissue, promotes insulin action, and has anti-inflammatory effects. Low levels of omentin are related to cardiovascular risk, insulin resistance, and obesity [18].

Chemerin

Adipogenesis, inflammation, and glucose homeostasis are regulated by chemerin. Chemerin levels are higher in obesity, T2DM, and metabolic syndrome [19].

Visceral vs. subcutaneous fat

The two primary types of adipose tissue are visceral adipose tissue (VAT) and subcutaneous adipose tissue (SAT). Internal organs are surrounded by VAT inside the abdominal cavity; SAT lies under the skin. VAT reduction interventions tend to be more effective than general fat loss because of VAT's significant link to metabolic disorders including insulin resistance and cardiovascular diseases. Insulin sensitivity improves while inflammation decreases and related condition risks reduce when VAT levels drop. Targeted reduction of VAT leads to superior metabolic health improvements compared to overall body fat loss. The metabolic profiles and adipokine secretion patterns of these fat depots differentiate them. VAT is metabolically active and contributes to systemic inflammation and insulin resistance by secreting increased amounts of pro-inflammatory adipokines, including tumor necrosis factor alpha (TNF-α) and interleukin-6 (IL-6) [20]. However, SAT releases adiponectin, which has anti-inflammatory and insulin-sensitizing properties [21]. The VAT-to-SAT ratio is related to increased metabolic risk, which includes cardiovascular disease and T2DM.

Muscle mass and its interaction with adipokines

Through its dual function as a movement provider and an endocrine organ that releases myokines which work alongside adipokines, skeletal muscle maintains metabolic homeostasis [22]. Research demonstrates that sufficient muscle mass enhances insulin sensitivity while adjusting adipokine profiles to produce elevated adiponectin levels and lower pro-inflammatory adipokine secretion. This interaction plays an important role in lessening metabolic disease risks including type 2 diabetes and cardiovascular disease [23]. Sarcopenia manifests through reduced muscle mass which produces worsened insulin resistance together with unfavorable adipokine secretion patterns that drive obesity-related metabolic disorders forward.

Effects of weight loss and gain on adipokine levels

Body weight fluctuations create direct changes in adipokine secretion which affects metabolic health. When weight gain occurs alongside increased VAT, individuals experience higher levels of pro-inflammatory adipokines and diminished adiponectin which leads to greater insulin resistance in addition to systemic inflammation [24]. When obese patients lose weight through exercise and dietary changes, they can improve their adipokine balances by reducing inflammatory proteins while boosting adiponectin levels [25]. These changes improve insulin sensitivity while lowering inflammation which leads to sustained metabolic advantages and lowers obesity-related disease risks.

Body composition and its impact on adipokine secretion

Adipokine secretion is influenced significantly by the adipose tissue distribution, muscle mass, and degree of weight change and thereby metabolic health. The impact of body composition on adipokine secretion is shown in Table 3.

**Table 3:** Impact of body composition on adipokine secretion VAT: Visceral adipose tissue; SAT: subcutaneous adipose tissue

Body Composition Factor	Effect on Adipokine Secretion	Metabolic Implications	Reference
Increased VAT	↑ Pro-inflammatory adipokines (IL-6, TNF-α) ↓ Adiponectin	↑ Inflammation ↑ Insulin resistance ↑ Cardiometabolic risk	[26]
Increased SAT	↑ Adiponectin ↓ Pro-inflammatory adipokines	↓ Inflammation ↑ Insulin sensitivity ↓ Cardiometabolic risk	[27]
Increased Muscle Mass	↑ Adiponectin ↓ Pro-inflammatory adipokines	↑ Insulin sensitivity ↓ Inflammation ↓ Metabolic risk	[22]
Weight Gain	↑ Pro-inflammatory adipokines ↓ Adiponectin	↑ Inflammation ↑ Insulin resistance ↑ Risk of metabolic diseases	[28]
Weight Loss	↓ Pro-inflammatory adipokines ↑ Adiponectin	↓ Inflammation ↑ Insulin sensitivity ↓ Risk of metabolic diseases	[29]

Molecular mechanisms underlying adipokine-mediated metabolic dysfunction

Bioactive molecules produced by adipose tissue function as adipokines which control metabolic activities. Obesity leads to abnormal adipokine production which establishes connections with chronic inflammation and insulin resistance along with additional metabolic diseases. Through an analysis of adipokine functions, this review examines their impact on metabolic dysregulation by evaluating their involvement in inflammatory processes as well as insulin signaling and hormonal system interactions to understand their multifaceted role in metabolic diseases.

Inflammatory pathways mediated by adipokines

**Figure 1:**
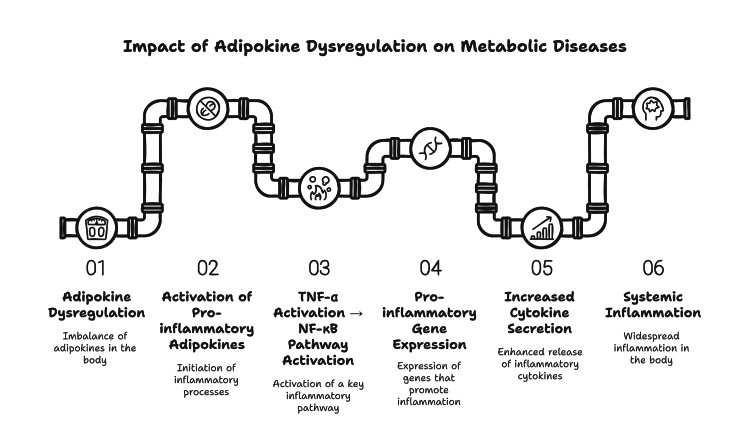
Impact of adipokine dysregulation on metabolic diseases Image credit: Author

Adipokine dysregulation and obesity are related to low-grade chronic inflammation. Not only is this inflammation confined within adipose tissue, but it is systemic and affects other organs and tissues. Key mediators of this inflammatory response are several adipokines, especially those secreted by VAT. Adipokines are proinflammatory, such as TNF-α, IL-6, and resistin, which participate in inflammation and activation of other inflammatory pathways that lead to insulin resistance and metabolic diseases. TNF-α stimulation of the nuclear factor-kappa B (NF-κB) pathway is one example, which downstream leads to pro-inflammatory gene expression. The consequence is an enhanced secretion of cytokines and chemokines that perpetuate systemic inflammation [4]. IL-6 triggers inflammatory cascades, inhibits insulin signaling, and promotes the "Janus Kinase/Signal Transducer and Activator of Transcription" (JAK/STAT) pathway [30]. In addition to disrupting insulin action in peripheral tissues, these inflammatory responses contribute to the increase of metabolic syndrome T2DM and cardiovascular diseases (Figure 1).

Adipokine influence on insulin signaling

Obesity and metabolic disease are defined by insulin resistance. Dysregulation of adipokines is centrally important in controlling insulin sensitivity and their dysregulation has a major impact on insulin signaling. One of the most studied adipokines in relation to insulin sensitivity is adiponectin, an anti-inflammatory adipokine. With the activation of AMP-activated protein kinase (AMPK) in the muscles and liver, adiponectin can increase insulin sensitivity by encouraging fatty acid oxidation and glucose absorption in the muscles and liver [31]. Adiponectin levels are reduced in obese individuals and insulin resistance, which indicates the protective role of adiponectin in ensuring glucose homeostasis.

In contrast, proinflammatory adipokines including TNF-α, resistin, and IL-6 also disrupt insulin signaling. For instance, insulin receptor substrate (IRS) proteins are serine phosphorylated by TNF-α, which starts the disintegration of the insulin signaling pathway and the emergence of insulin resistance. Resistin has been shown to inhibit insulin's ability to enhance glucose absorption in adipocytes and myocytes, hence reducing insulin sensitivity. In addition, IL-6 acts on insulin signaling through inflammatory pathways that activate the suppression of normal insulin receptor function [11]. Table 4 depicts the effects of adipokines on insulin signaling.

**Table 4:** Adipokines and their effects on insulin signaling JAK/STAT: Janus Kinase/Signal Transducer and Activator of Transcription

Adipokine	Effect on Insulin Signaling	Mechanism	Reference
Adiponectin	Enhances insulin sensitivity	Activates AMPK to promote glucose uptake and fatty acid oxidation	[32]
TNF-α	Impairs insulin signaling	Disrupts insulin signaling by causing IRS-1 to become serine phosphorylated.	[33]
Resistin	Reduces insulin sensitivity	Reduces the absorption of insulin-stimulated glucose by myocytes and adipocytes.	[34]
IL-6	Impairs insulin sensitivity	Activates JAK/STAT and NF-κB pathways that interfere with insulin signaling	[35]

The interrelationship between adipokines and hormonal systems

Adipokines do not work in isolation. They interact with other hormonal systems to control metabolic processes. The "hypothalamic-pituitary-adrenal" axis, which controls stress responses, has a crucial relationship with adipokines. It has been shown that cortisol, a stress-induced hormone, influences adipokine secretion. Obesity involves elevated levels of cortisol that indirectly encourage the secretion of TNF-α and IL6, two adipokines that cause inflammation, which increases metabolic dysfunctions [36].

Adipokines also have another important interaction with the autonomic nervous system (ANS). Adipokine secretion is influenced by catecholamines released from the ANS. One example is the release of norepinephrine from sympathetic nerve fibers altering adiponectin and resistin secretion [37]. The sympathetic nervous system is often overactive in individuals with obesity and this dysregulated adipokine profile contributes to metabolic dysfunction.

**Figure 2:**
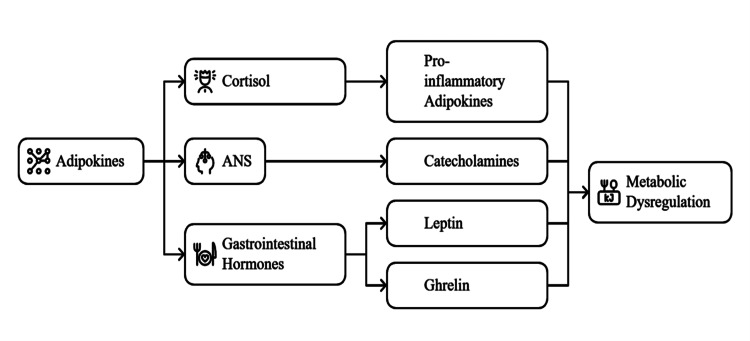
Hormonal interactions mediated by adipokines in metabolic regulation Image credit: Author ANS: Autonomic nervous system

Additionally, adipokines also interact with gastrointestinal hormones, like ghrelin and leptin. Adipocytes produce leptin, and it is known to regulate appetite and energy balance. It tells the hypothalamus to reduce hunger and burn more energy. Despite this, however, in obesity leptin resistance occurs with leptin failing to regulate these processes, resulting in overeating and weight gain [28]. Interactions with adipokines are key to ghrelin's role in appetite and energy balance regulation, but ghrelin secretion is disrupted in obesity and contributes to metabolic dysregulation (Figure 2) [38].

Clinical implications and therapeutic potential

Our understanding of obesity and related diseases has not only progressed but also provided many clinical implications of the intricate relationship between adipokines and metabolic diseases. Adipokines are ideal biomarkers for metabolic diseases and targeting adipokine pathways may be a new therapeutic strategy. In addition, lifestyle interventions, including diet and exercise, affect adipokine profiles that can be used to affect metabolic health.

Adipokines as Biomarkers for Metabolic Diseases

Adipokines play a progressively important role as biomarkers for the analysis, prognosis, and therapy of metabolic disorders, including obesity, T2D (Type 2 diabetes), and cardiovascular diseases. Given their roles in regulating metabolic processes such as insulin sensitivity, inflammation, and lipid metabolism, they are ideal candidates to reflect the underlying pathophysiology of these conditions. For example, adiponectin, a well-established biomarker of insulin sensitivity, is inversely correlated with visceral fat. Adiponectin is being used as a diagnostic tool to predict T2DM and metabolic syndrome since it is associated with obesity and insulin resistance [39]. Likewise, leptin, a marker of fat mass that regulates appetite and energy balance, can be elevated in obesity and be a marker of leptin resistance (increased appetite and weight gain) (Table 5) [40].

**Table 5:** Adipokines as biomarkers for metabolic diseases

Adipokine	Role in Metabolic Disease	Clinical Implication	Reference
Adiponectin	Inversely correlated with insulin resistance and obesity	Potential biomarker for diagnosing insulin resistance and metabolic syndrome	[39]
Leptin	Reflects fat mass, elevated in obesity, associated with leptin resistance	Can be used as a marker for obesity-related metabolic disturbances	[40]
Resistin	Linked to insulin resistance and inflammation	Elevated levels correlate with cardiovascular risk and insulin resistance	[10]
TNF-α	Pro-inflammatory, linked to insulin resistance	Elevated levels are associated with obesity, diabetes, and cardiovascular disease	[41]

In contrast, resistin is a pro-inflammatory adipokine related to cardiovascular risk factors and insulin resistance. It is elevated in people with obesity and may be a marker of metabolic dysfunction. Resistin levels have also been found to be elevated in association with a high risk of atherosclerosis and other cardiovascular diseases [10]. Early detection of metabolic disturbance and stratification of patients for personalized intervention can be enabled by measuring adipokines such as adiponectin, leptin, resistin, and TNF-α [41].

Targeting Adipokine Pathways: Treatment Strategies

Given the critical role of adipokines in metabolic dysregulation, therapeutic strategies targeting adipokine pathways are under investigation as potential treatments for obesity, and obesity-associated metabolic diseases, such as insulin resistance [42].

Another promising therapeutical target is resistin antagonists. It may be possible to reduce insulin resistance and improve glucose metabolism by "weakening" resistin's pro-inflammatory effects. Several studies have shown that blocking resistin decreases insulin sensitivity in animal models [43]. Additionally, TNF-α inhibitors have been tested to attenuate the inflammatory burden in obesity and improve insulin sensitivity and prevent progression of metabolic diseases. Obese individuals have been shown to have reduced markers of inflammation and improved metabolic parameters with anti-TNF-α therapies (such as infliximab) [44].

While promising, these targeted therapeutic strategies are yet to reach the clinical stage, with further clinical trials necessary to determine their safety and efficacy in humans.

Lifestyle Interventions: Adipokine Profiles

**Figure 3:**
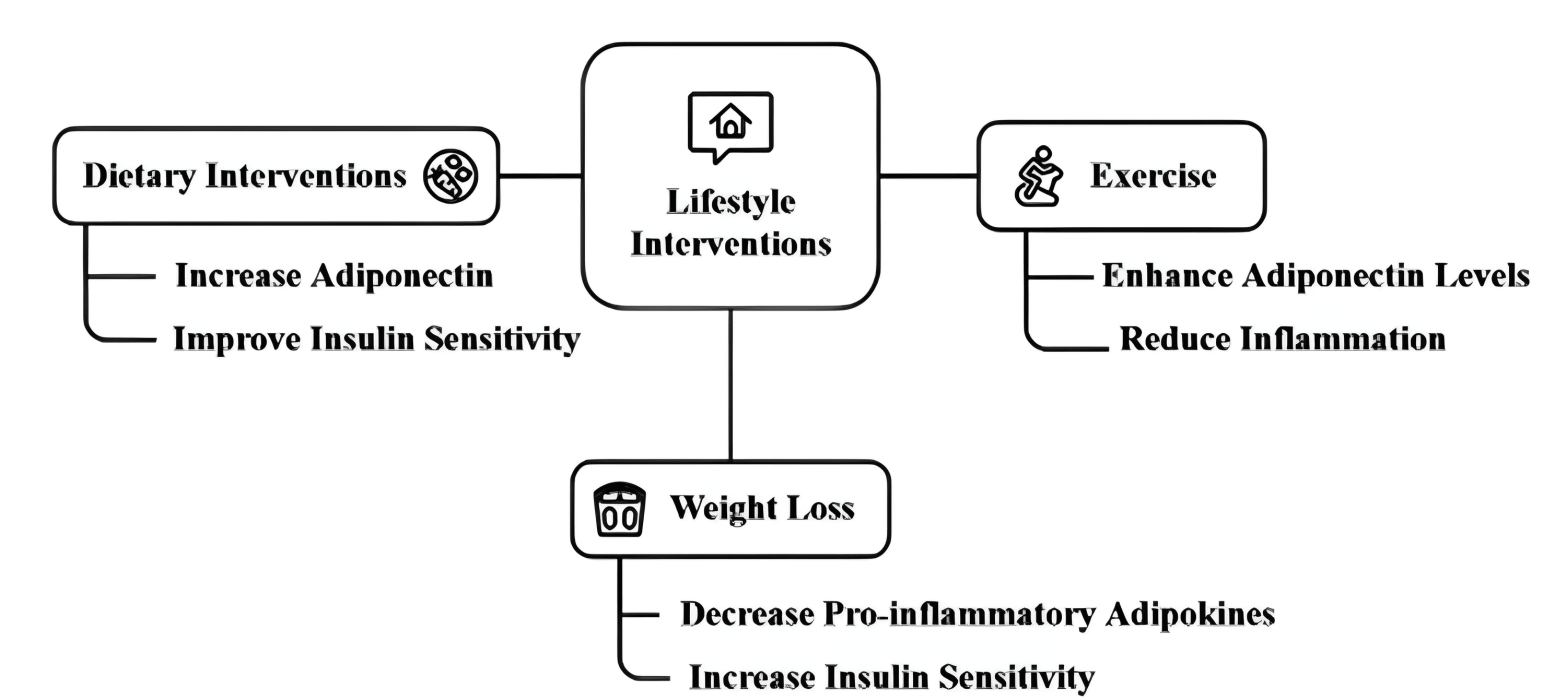
Effects of lifestyle interventions on adipokine profiles Image credit: Author

The effects of dietary adaptations and exercise on adipokine secretion exist but this information does not directly support our treatment hypothesis which investigates adipokine functions in metabolic regulation. We have discussed how adipokine levels respond to physiological changes from weight loss and weight gain because these responses affect insulin sensitivity and metabolic health. Our study should focus on demonstrating how weight-induced changes in adipokine patterns help regulate metabolic disease processes. The discussion of calorie restriction and exercise should become part of total weight management strategies instead of existing as separate methods (Figure 3).

Dietary Interventions

Studies have indicated that diets rich in omega-3 fatty acids, low in saturated fats, and high in fiber raise adiponectin levels enhance insulin sensitivity and lower inflammation. Conversely, adiponectin levels are reduced and proinflammatory adipokines such as TNF-α and IL6 are increased by high-fat diets, especially those high in trans fats [45].

Exercise

The other potent modulator of adipokine profiles is regular physical activity. Adiponectin levels can be increased by both aerobic and resistance exercise, and both increase insulin sensitivity and decrease the secretion of pro-inflammatory adipokines. Exercise has been shown to reduce body fat, especially visceral fat, with reductions in body fat associated with improved adipokine profiles and reduced metabolic risk [46]. On top of that, exercise promotes the gain in muscle, which in turn regulates the secretion of myokines and adipokines and better metabolic health.

Weight Loss

Normal adipokine levels have been shown to be restored with weight reduction, either through bariatric surgery or intensive lifestyle interventions. In fact, high weight loss increases adiponectin levels and decreases proinflammatory cytokines, which leads to increased insulin sensitivity and decreased metabolic syndrome parameters [47].

Current challenges and future directions

Research confirms that adipokines function as essential controllers of metabolic processes while serving as fundamental factors in obesity-related metabolic disease development. The absence of standardized assays to measure adipokines represents a fundamental obstacle in adipokine research because it restricts both the uniformity of results and their ability to be compared between different studies. Creating international guidelines together with calibration protocols represents a necessary step toward solving this measurement standardization problem. Standardized assays for adipokine measurement would produce accurate and reliable data collection which supports clinical application development. Moving forward research needs to concentrate on developing better measurement techniques alongside enhanced sensitivity and specificity as well as standardized protocols throughout both research laboratories and clinical environments. This review summarizes adipokines' therapeutic effects but recognizes that standardized measurement practices are essential for basic research progression and clinical application improvement.

Limitations in adipokine research

Although research on adipokines has been extensive, researchers still face significant limitations that prevent a complete understanding of adipokine regulation of metabolism and its role in disease processes. The main difficulty with adipokines lies in their diverse nature as bioactive molecules which both share functions and engage in complex interactions thereby complicating the identification of their exact impact on metabolic diseases. The extensive variation of adipokine levels between different populations and across genders and age groups creates difficulties in using them as dependable biomarkers for clinical applications. The concentrations of adipokines show major variations between different ethnic groups which complicates the creation of standard reference ranges or cutoffs. The variable nature of adipokines reduces their effectiveness as independent diagnostic tools for disease detection [47]. A more complete diagnostic approach emerges from pairing adipokine profiles with additional metabolic markers including glucose measurements alongside insulin and lipid profiles. The integrated approach offers a solution to adipokine level variability which enables more precise metabolic health assessments [48].

The review examines measurement difficulties for adipokines while demonstrating their possible therapeutic value which demands additional research and methodological improvements. This review presents the existing knowledge about adipokines and suggests research directions for future study while making clear that adipokine variability and complexity present opportunities for deeper research that will improve clinical applications.

Adipokine research faces challenges because scientists cannot determine direct causal links between adipokines and metabolic diseases. The observational nature of most current studies prevents researchers from reaching definitive conclusions about adipokine roles in the pathophysiology of cardiovascular disease, obesity, and insulin resistance. Würfel et al. [49] explain that adipokines represent possible treatment targets for obesity despite our incomplete understanding of their mechanisms of action on metabolic health. The clinical application of adipokines faces complexity because individual responses to these signaling proteins vary widely [49].

Personalized medicine approaches

Growing research evidence demonstrates how adipokines connect to metabolic diseases which positions them as promising biomarkers for advancing personalized medicine. Due to the variable adipokine levels among different populations and individuals, clinicians must establish frameworks that merge adipokine profiling with additional diagnostic methods to create effective treatment plans. Developing algorithms to merge adipokine measurements and genetic and metabolic alongside clinical data can provide complete metabolic health profiles for patients. Scientists may find anti-inflammatory treatments useful for patients who present raised levels of TNF-α or resistin pro-inflammatory adipokines to restore normal function within adipokine systems. The accuracy of medical diagnosis and treatment plans improves when personalized algorithms take into account variables like ethnicity, gender, and age along with clinical markers such as glucose and lipid profiles. An integrative strategy enables healthcare professionals to bypass current adipokine variability challenges which leads to better designed personalized treatment plans for patients. Although more evidence and validation are necessary, adipokine profiling together with other metabolic markers shows significant potential to propel personalized medicine forward.

Targeted therapies that modulate adipokine levels could also be part of personalized medicine. For instance, receptor agonists or gene therapy may be used to elevate adiponectin levels in individuals suffering from metabolic syndrome or T2DM. Likewise, leptin-based therapies may be beneficial in people with severe obesity and leptin resistance. Lifestyle interventions such as custom-designed dietary plans or personalized exercise programs could be included, based on the principle of tailoring these to adjust adipokine profiles in a way that promotes metabolic health.

Nevertheless, in order to realize personalized medicine strategies based on adipokines, we need to further elucidate the individual adipokine responses and develop precision medicine tools to measure the metabolic status of patients with accuracy. 

Emerging technologies in adipokine measurement

The precision of adipokine measurement has advanced through recent technological developments including mass spectrometry and single-cell RNA sequencing (scRNA-seq). Multiple adipokines undergo simultaneous accurate analysis through mass spectrometry while scRNA-seq provides detailed cellular-level expression insights. Improved diagnostic precision from recent developments enables adipokines to serve as dependable biomarkers that support early disease discovery and individualized therapeutic approaches [46]. Limitations of traditional methods of adipokine measurement include low sensitivity, low specificity, and low throughput. However, mass spectrometry (MS) has become a more precise and higher throughput tool for quantifying adipokines and profiling their molecular forms. MS-based techniques can identify novel adipokines and measure their isoforms, which may provide novel insight into their function and their role in metabolic disease [50].

Biosensor development for real-time adipokine monitoring is another emerging technology. Continuous monitoring of patients' adipokine concentrations could be enabled by these sensors, enabling personalized treatment adjustments. The development of portable, cost-effective devices for adipokine measurement is being enabled by recent advances in microfluidics and lab-on-a-chip technology. The potential of these technologies to dramatically transform how adipokine profiles are monitored could mean frequent, accurate measurements are possible.

The combination of MS with scRNA-seq represents a transformative opportunity for adipokine-related diagnostic methodologies. Through MS, scientists achieve accurate adipokine measurements which enable them to construct comprehensive profiles for early disease detection and treatment assessment. The simultaneous analysis capability of this method enhances diagnostic accuracy by measuring multiple adipokines at once [50]. The examination of cellular heterogeneity helps researchers understand adipokine regulation of metabolism. These combined technologies enable personalized medical approaches while advancing disease monitoring capabilities and expanding knowledge about metabolic conditions [51].

## Conclusions

The review examines how adipokines regulate metabolic diseases by affecting critical body functions including inflammation and insulin sensitivity along with energy metabolism. Despite substantial advances in deciphering their mechanisms of action scientists face ongoing obstacles because of their intricate nature and variable characteristics alongside the challenge of proving direct disease connections. The adipokines adiponectin, leptin, and resistin serve essential functions in the development of obesity and cardiovascular diseases as well as insulin resistance. Non-pharmacological disease management strategies emerge from diet and exercise interventions that demonstrate capability to adjust adipokine levels. Mass spectrometry together with biosensors and single-cell RNA sequencing represent breakthrough tools that enhance diagnostic accuracy and therapeutic monitoring capabilities. The progress achieved so far requires additional research to solve current problems including better measurement accuracy of adipokines and understanding their various functions in different populations. Overcoming present research limitations demands the establishment of standardized measurement protocols alongside longitudinal studies. Successful adipokine research progress and targeted therapeutic development depend on interdisciplinary partnerships among endocrinologists, data scientists, and bioengineers. Such research activities will establish foundations for developing improved medical interventions against obesity and its metabolic complications.
